# Transmission of West Nile Virus during Horse Autopsy

**DOI:** 10.3201/eid1603.091042

**Published:** 2010-03

**Authors:** Marietjie Venter, Johan Steyl, Stacey Human, Jacqueline Weyer, Dewald Zaayman, Lufcille Blumberg, Patricia A. Leman, Janusz Paweska, Robert Swanepoel

**Affiliations:** University of Pretoria, Pretoria, South Africa (M. Venter, J. Steyl, S. Human, D. Zaayman); National Institute for Communicable Diseases, Sandringham, South Africa (M. Venter, J. Weyer, L. Blumberg, P.A. Lehman, J. Paweska, R. Swanepoel)

**Keywords:** West Nile virus, encephalitis, transmission, horses, zoonotic infection, viruses, South Africa, letter

**To the Editor:** West Nile virus (WNV) circulates mainly in birds and ornithophilic mosquitoes. Humans and horses are considered incidental, dead-end hosts (*1*). Fever, rash, arthralgia, and myalgia develop in ≈20% of cases in humans; severe neurologic disease may develop in <1% (*1*). In horses, 20% of infections result in clinical disease, of which ≈90% involve neurologic disease with ataxia, weakness, recumbency, muscle fasciculation, and high death rates (30%) (*2*).

Genetic variants of WNV include lineage 1 found in the Northern Hemisphere and Australia; lineage 2 found mainly in southern Africa and Madagascar (*3*); lineages 3 and 4 found in central and eastern Europe (*4*); and lineage 5 found in India (*5*). Differences in neuroninvasiveness and pathogenic potential are functions of individual genotypes, not lineage (*3*,*6*–*8*).

We recently reported WNV lineage 2 in several cases of neurologic disease in horses in South Africa (most cases were fatal) (*7*). We report a case of zoonotic transmission to a veterinary student during the autopsy of a horse. The study was reviewed and approved by the Ethics Committee of the University of Pretoria, and informed consent was provided by the veterinary student.

On April 9, 2008, a 4-month-old Welsh pony from Gauteng in South Africa had fever, Schiff-Sherrington signs, and a leukocyte count of 32 × 10^9^ cells/L. He was treated with dimethyl sulfoxide, dexamethasone, and chloramphenicol and responded well. He was able to stand with help, and did not show neurologic signs at this stage. On May 9, he was sent home and was able to walk with support. On May 12, he had a relapse with neurologic deterioration and rectal prolapse, and was treated with antiinflammatory agents. Symptoms worsened and he was humanely killed on May 15 by using ketamine and MgSO_4_. The carcass was sent to the Faculty of Veterinary Sciences, University of Pretoria, for autopsy because of unusual neurologic signs in the pony. Autopsy was performed by a veterinary pathologist and 2 students on May 16, 2008.

Macroscopic findings included moderate intermuscular, fascicular, perineural edema, severe diffuse pulmonary edema, mild hydropericardium, and rectal prolapse resulting in marked submucosal edema and mucosal hyperamia, i.e., traumatic proctitis. The spinal cord up to C1 showed marked Wallerian degeneration of the peripheral white matter from the median fissure, which extended along the ventral funiculus up to the most dorsal section of the lateral funiculus. Changes were characterized by white matter spongiosis with numerous digestion chambers containing phagocytosing myelinophages and scattered interstitial gemistocytes. No inflammatory reaction was detected. We also observed septal edema and moderate multifocal perivascular and peribronchiolar lymphocytic infiltration with occasional apoptosis in the lungs.

The brain, which was removed by 1 of the students, was sent to the Department of Medical Virology, University of Pretoria, for WNV reverse transcription–PCR (RT-PCR). The lungs were sent to Onderstepoort Veterinary Institute for African horse sickness RT-PCR. WNV-specific real-time RT-PCR showed positive results. DNA sequencing and phylogenetic analysis identified WNV lineage 2 in several sections of the brain (HS23/2008, GenBank accession no. FJ464376). Results of African horse sickness RT-PCR on lung tissue specimens were inconclusive and could not be confirmed by culture.

On May 22, six days after the autopsy on the horse, fever, malaise, myalgia, stiff neck, and severe headache developed in the veterinary student who had handled the horse brain. A rash appeared 2 days later. Symptoms persisted for ≈10 days. The patient was treated symptomatically by an infectious disease specialist and prescribed bed rest. Because cases of Rift Valley fever were recently reported in veterinarians in South Africa, serum was sent to the National Institute for Communicable Diseases, where a virus isolate was obtained in suckling mice and identified as WNV by RT-PCR.

After diagnosis of WNV infection in the pony, RNA extracted from the original human serum and from the suckling mouse isolate was sent to the Department of Medical Virology, University of Pretoria, for DNA sequencing and phylogenetic analysis of the virus. Comparison of part of the nonstructural protein 5 gene identified identical sequences from the student’s serum, the virus isolate, and the pony’s brain. All sequences clustered with lineage 2 WNV and were closely related to isolates obtained from horses diagnosed with fatal WNV encephalitis in South Africa in 2008 (*7*) (Figure).

**Figure:**
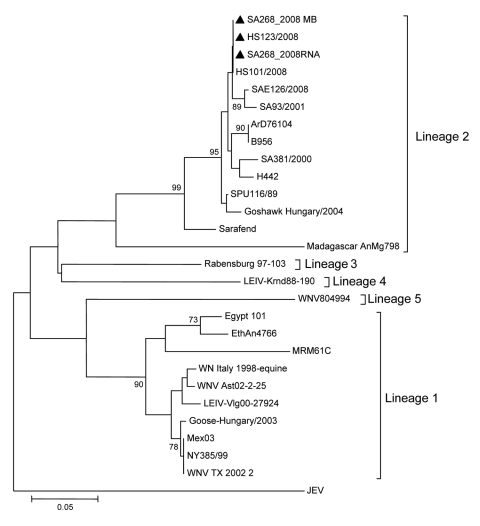
Phylogenetic comparison of West Nile virus (WNV) nonstructural protein 5 partial gene fragment identified in a veterinary student’s serum and in the virus isolate obtained from mouse brain and the horse’s brain after autopsy (triangles) relative to other WNV strains from South Africa and elsewhere. The neighbor-joining tree was compiled by using MEGA version 4 software (www.megasoftware.net/under) and 1,000 bootstrap replicates by using the maximum composite likelihood algorithm. Genetic lineages are indicated on the right as described (*3*–*7*). Scale bar indicates nucleotide substitutions per site. JEV, Japanese encephalitis virus (included as outgroup).

Human infections with WNV have been described after bird autopsies and needle stick injury in laboratory workers (*9**,**10*). The case acquired by our patient suggests a zoonotic risk exists for infection with WNV during autopsy of horses that died from neurologic disease. Although humans and horses are considered to have low-grade viremia, virus levels may be higher in nerve tissue.

The patient wore latex gloves, his only protection during the autopsy, and had removed the spinal cord and brain. No protective inhalation or eye equipment was worn. No autopsy assistants or other students who worked with or were near the carcass became sick or seroconverted. The most likely route of infection may have involved exposure of mucous membranes to droplets. After the incident, biosafety measures were improved and included wearing of masks and eye protection gear during autopsies at the facility.
